# Exposure and Risk Assessment to Airborne dl-PCBs and Dioxins in the Population Living in the Neighborhood of a Cement Plant: A Pilot Study in the Valencian Region of Spain

**DOI:** 10.3390/toxics11040389

**Published:** 2023-04-20

**Authors:** Pablo Ruiz, Iñaki Lacomba, Antonio López, Vicent Yusà, Clara Coscollà

**Affiliations:** 1 Public Health Laboratory of Valencia, 21, Avenida Catalunya, 46020 Valencia, Spain; 2 Foundation for the Promotion of Health and Biomedical Research in the Valencian Region, FISABIO—Public Health, 21, Avenida Catalunya, 46020 Valencia, Spain; lacomba_ina@gva.es (I.L.); vicent.yusa@fisabio.es (V.Y.); coscolla_cla@gva.es (C.C.)

**Keywords:** dioxins, ambient air, risk assessment, cement plant, cancer risk

## Abstract

Emissions from cement manufacturing facilities may increase health risks in nearby populations. For this reason, dioxin-like PCB (dl-PCB), polychlorinated dibenzo-p-dioxin (PCDD), and polychlorinated dibenzofuran (PCDF) concentrations in PM_10_ samples were assessed in the vicinity of a cement manufacturing plant located in the Valencian Region (eastern Spain). The total concentrations of the sum of dl-PCBs, PCDDs, and PCDFs ranged between 1.85 and 42.53 fg TEQ/m^3^ at the assessed stations. The average daily inhalation dose (DID) for the sum in adults ranged from 8.93 · 10^−4^ to 3.75 · 10^−3^ pg WHO TEQ kg^−1^ b.w. d^−1^, and, for children, the DID ranged from 2.01 · 10^−3^ to 8.44 · 10^−3^ pg WHO TEQ kg^−1^ b.w. d^−1^. Risk assessment for adults and children was performed using both daily and chronic exposure. The hazard quotient (HQ) was calculated considering 0.025 pg WHO TEQ kg^−1^ b.w. d^−1^ to be the acceptable maximum permitted inhalation exposure. The HQ obtained was slightly higher than 1 for PCDD/Fs at one of the stations (Chiva), indicating a possible health risk for the population under study due to inhalation exposure. In the case of chronic exposure, cancer risk (>10^−6^) was observed for some samples in one of the assessed sampling sites (Chiva).

## 1. Introduction

Cement production has increased around the world as a consequence of the growth of population and urbanization ratio [1]. Dioxins, furans (PCDD/Fs), and dioxin-like PCBs (dl-PCBs) can be produced in the cement plant due to the fuel or to its synthesis in the gas treatment system [2]. Combustible material introduced in the precalcination zone can react with the chlorine not retained by the alkaline matrix of the clinker producing dl-PCB and dioxin emissions [3].

Dl-PCBs and PCDD/Fs are regulated and are part of the “dirty dozen” (the 12 persistent organic pollutants regulated by the Stockholm Convention) [4]. Their prolonged exposure can cause reproductive, endocrine, and immunological disorders [5,6,7]. The impact and exposure of dl-PCBs and dioxins is a concerning matter because people are exposed to these contaminants for large periods of their lives.

Although many studies have focused on knowing the emissions of PCDD/Fs and PCBs from cement industries by sampling directly inside the stack gas [8,9], there is little written about the exposure of populations close to the cement industry. Breathing dl-PCBs and dioxins airborne in polluted ambient air around locations close to hotspots such as cement plants could be a relevant exposure source in nearby populations. According to the World Health Organization, the daily inhalation exposure can be considered to be 10% of the tolerable daily intake (TDI) by ingestion exposure if we consider the worst-case scenario [10]. Ingestion exposure is considered the main source of dioxin and dl-PCB exposure in non-hotspot areas [11].

Previous risk assessment studies have studied PCDD/Fs in the ambient air of populations living close to cement plants [12,13,14,15]. Nevertheless, no study has investigated the exposure to dl-PCBs in the population located close to cement plants.

Previous studies have shown the population exposure to dl-PCBs and PCDD/Fs in the Valencian Region (Spain). Quijano et al., 2018 [16] observed that the average adult’s daily intake through diet was 1.58 pg TEQ (toxic equivalent) kg ^−1^ b.w. day ^−1^, and the average for children was 2.76 pg TEQ kg ^−1^ b.w. day ^−1^. Daily inhalation exposure to dl-PCBs and PCDD/Fs has been assessed by López et al., 2021 [17], observing a maximum average inhalation exposure of 1.08 · 10^−2^ pg TEQ kg^−1^ b.w. day^−1^ in adults and 2.42 · 10^−2^ pg TEQ kg^−1^ b.w. day^−1^ in children.

The exposure of lactating mothers and breastfed infants to PCDD/Fs and dl-PCBs was below the reference level reported by the EFSA (5.9 pg TEQ g^−1^) [18], but this value was exceeded when the 95th percentile or the maximum level was considered in a study carried out in the same region in breast milk samples [19]. Consequently, exposure of these compounds in infants, children, and adults is relevant. It is important to highlight that these substances can produce endocrine, reproductive, neurological, and immunological diseases and, finally, decrease life expectancy [20].

Due to the scarce international information about the influence of cement manufacturing emissions on inhalation risk assessment for dl-PCBs, the present work is a pilot study that has focused on measuring dl-PCB, PCDD, and PCDF profile congeners close to a cement plant located in the Valencian Region (Spain) and assessing the potential human health risks in nearby populations.

## 2. Materials and Methods

### 2.1. Reagents and Chemicals

High-purity reagents dichloromethane, n-hexane, and toluene and HPLC grade acetone were supplied by Merck (Taufkirchen, Germany). 1 µm PTFE filters were supplied by Pall Corporation (South Wagner Road, USA). Columns for sample preparation such as CLDS-ABN-STD (silica), CLDA-BAS-011 (alumina), and CLDC-CCE-034 (carbon) were supplied by Fluid Management System (Watertown, MA, USA) and glass wool for filtration from Merck (Taufkirchen, Germany). Nonane puriss standard for GC was supplied by Merck (Taufkirchen, Germany). The 17 high-purity certified commercial standards of 2,3,7,8-chloro-subsituted PCDD and PCDF and the 12 dl-PCBs congeners, as well as surrogates (EPA1613-LCS and WP-LCS) and internal standards (EPA1613-ISS and WP-ISS), were purchased from Wellington Laboratories (Guelph, ON, Canada).

### 2.2. Sampling and Site Characterization

Samples were collected, as a pilot study, in two towns (Chiva and Buñol) located close to a cement plant in different periods during the year 2020. The S1 sampler was placed in the town of Chiva (14,941 inhabitants) located to the east of the cement plant and 289 m above sea level. Thirteen PM_10_ samples were collected at this station from February to June 2020.

**Figure 1:**
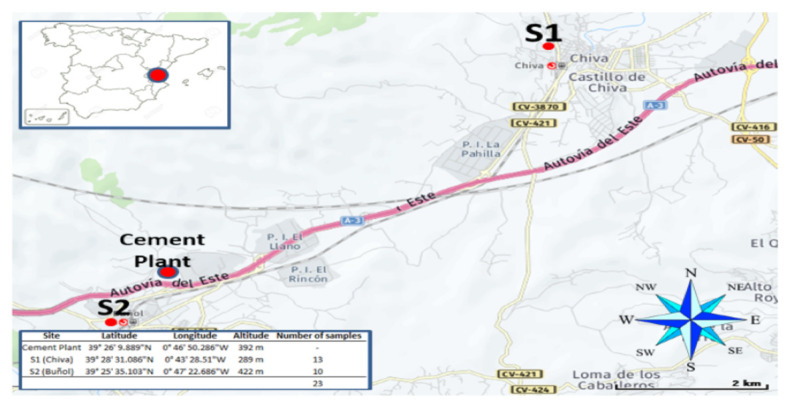
Location of the evaluated stations (S1 and S2) and cement plant.

The S2 sampler was placed in the town of Buñol (9517 inhabitants), located to the west of the cement plant. Ten PM_10_ samples were collected from August to October 2020 at this station (see Figure 1).

Air samples were taken as described in López et al. (2021) [17], using a high-volume sampling device (HVS) from Digitel (DPA-80, Volketswill, Germany). Total sampling volumes were around 1500 m^3^ (see Supplementary Table S1) with a sampling flow of 30 m^3^ h^−1^ for 48 h. For this, 150 mm diameter quartz fiber filters (QFF) were employed, which were supplied by Munktell Filter AB (Falun, Sweden). Samplers belong to the monitoring network of the Regional Valencia Government (Generalitat Valenciana).

### 2.3. Analytical Methodology and Identification Criteria

Sample preparation and GC-HRMS system were previously described by López et al. (2021) [17].

Identification and confirmation criteria were based on Commission Regulation 2017/771 [21] and EPA method 1613 [22] (see Supplementary Section S1).

For the calculation of the LOQ (quantification limit), the “Guidance Document on the Estimation of LOD (detection limit) and LOQ (quantification limit) for Measurements in the Field of Contaminants in Feed and Food” [23] was employed. The quantification limit for each sample in PM_10_ was calculated using TargetQuan software, and the average for the ∑PCDD/Fs and ∑dl-PCBs were 0.43 fg TEQ/m^3^ and 0.03 fg TEQ/m^3^, respectively.

### 2.4. Quality Control Protocol

Each batch of samples included a blank field that was treated in the same way as the air samples. In addition, quality control was carried out using a spiked blank every two analytical sets, and surrogates were evaluated in each sample. Filter analysis was performed after sampling or after the storage period at −20 °C.

### 2.5. Gaseous-Particle Partitioning Model

To estimate the total air concentrations from the obtained results for PM_10_, the model validated previously by López et al. (2021) [17] was used, in which the fraction of the compound in the particle phase (ø) and gas phase (1−Ø) was calculated using the Harner–Bidleman absorption model [24]:

(1) $$Ø=\frac{K_{p}\cdot C_{TSP}}{1+K_{p}\cdot C_{TSP}}$$

where *C_TSP_* is the concentration of total suspended particles in the air. According to Yusà et al. (2014) [24], its value is 55 µg m^−3^ in the assessed region, and K_p_ (m^3^ µg^−1^) is the gas-particle partition coefficient; its value can be calculated using the Equation (2):

(2) $$\mathrm{logKp}={\mathrm{logK}}_{oa}+\log f_{OM}-11.91$$

where log *f_OM_* is the fraction of organic matter that was calculated assuming a *f_OM_* of 0.2 as representative values in the studied region (Yusà et al., 2014) [25]. Log K_oa_ is the octanol–air partition coefficient, which is different for PCDD/Fs and dl-PCBs. For PCDD/Fs, K_oa_ is a function of the retention time indices (RTI) and temperature (*T*). The employed formula is different for tetra- to hexa-chlorinated PCDD/Fs (3) and for other PCDD/Fs (4) [24]:

(3) $${\mathrm{logK}}_{oa}=\frac{986}{T}+0.55+\frac{1.714}{T}\cdot \mathrm{RTI}-0.0032\cdot \mathrm{RTI}\;$$

(4) $${\mathrm{logK}}_{oa}=\frac{1672}{T}+2.98+\frac{0.857}{T}*\mathrm{RTI}-7\cdot 10^{-5}\cdot \mathrm{RTI}\;$$

Dl-PCBs K*_oa_* was calculated using the following Equation (5) [26]:

(5) $${\mathrm{logK}}_{oa}=K_{ow}+\log\frac{\mathrm{RT}}{H}$$

where *K_OW_* is the octanol–water coefficient for each analyte, T is the temperature (295 K), R is the gas constant (8.21 · 10^−5^ atm m^3^ mol^−1^ K^−1^), and *H* is the Henry coefficient (atm m^3^ mol^−1^).

### 2.6. Exposure and Risk Assessment

Based on the risk assessment protocol for human health [27], both (a) daily inhalation exposure and (b) lifetime exposure to PCDD/Fs and dl-PCBs were considered:

(a) In order to calculate the daily intakes, the daily inhalation dose (DID) was estimated for both adults and children (<6 years):

(6) $$\mathrm{DID}=\frac{C\cdot \mathrm{IR}\cdot \mathrm{ET}}{\mathrm{BW}}\;(\mathrm{pg}\;\mathrm{WHO}\;\mathrm{TEQ}\;{\mathrm{kg}}^{-1}\;b.w.d^{-1})$$

where C is the total ambient air concentration of PPCDD/F and dl-PCB using upper limit concentrations in pg TEQ/m^3^; IR is the inhalation rate per hour (0.83 m^3^ h^−1^ for adults and 0.4 m^3^ h^−1^ for children); ET is the exposure time (24 h); and BW is the mean body weight (70 kg for adults and 15 kg for children) [28].

To estimate the risk, the recommended tolerable daily intake (TDI) (0.25 pg TEQ kg^−1^ day^−1^ for PCDD/Fs plus dl-PCBs) was taken as reference, according to the EFSA [18].

In order to put this exposure (DID) in a risk assessment context, a hazard quotient (HQ) was also calculated, considering:

(1) The DID value in each station as p95.
(2) Considering that daily inhalation exposure accounts for around 10%, this percentage of TDI as a reference health-based value was employed (Equation (7)) [29].
  (7) $$\mathrm{HQ}=\frac{\mathrm{DID}\;\left(p95\right)}{0.1\cdot \mathrm{TDI}}$$

(b) To calculate the risk of cancer due to lifetime exposure, the product of chronic exposure (CE) and the slope factors (SF) was used [30].

Cancer risk= CE·SF (8)

Cancer exposure was estimated by multiplying the total concentration of each compound analyzed in mg m^−3^ (C _air_) and the inhalation factor (IF) (Equation (9)), obtained from Equation (10):

CE = C_air_ · IF (mg kg^−1^ day^−1^) (9)

(10) $$\mathrm{IF}=\frac{{\mathrm{IR}}_{\mathrm{inh}}\cdot \mathrm{EF}\cdot \mathrm{ED}\cdot \mathrm{ET}}{\mathrm{BW}\cdot \mathrm{AT}}\;(m^{-3}\;{\mathrm{kg}}^{-1}\;{\mathrm{day}}^{-1})$$

where IR_inh_ is the inhalation rate per day (20 m^3^ day^−1^); EF is the exposure frequency (365 days) per year; ED is the exposure duration (70 years); ET is the exposure time (24 h day^−1^); BW is the body weight of the subject (70 kg); and AT is average length of time of carcinogenic exposure over a lifetime (25,500 days = 70 years × 365 days per year) [29]. Using these parameters and the Inhalation Risk Units (IUR) of each contaminant, which represent the cancer potency factor for inhalation exposure [30], the SF was calculated according to Equation (11). The IUR values used are shown in Table S2.

(11) $$\mathrm{SF}\;{\left(\frac{\mathrm{mg}}{\mathrm{kg}\;{\mathrm{day}}^{-1}}\right)}^{-1}=\frac{\mathrm{IUR}\;(\frac{µg}{m^{3}}{)}^{-1}\cdot \mathrm{BW}\;(\mathrm{kg})\;\cdot \;1000\;(\frac{µg}{\mathrm{mg}})}{{\mathrm{IR}}_{\mathrm{inh}}\;}$$

The cancer risk values were compared to the carcinogenic benchmark level, considering values higher than 10^−6^ as concerned cancer risk and values higher than 10^−4^ as unacceptable [27].

## 3. Results and Discussion

### 3.1. Total Concentrations

**Figure 2:**
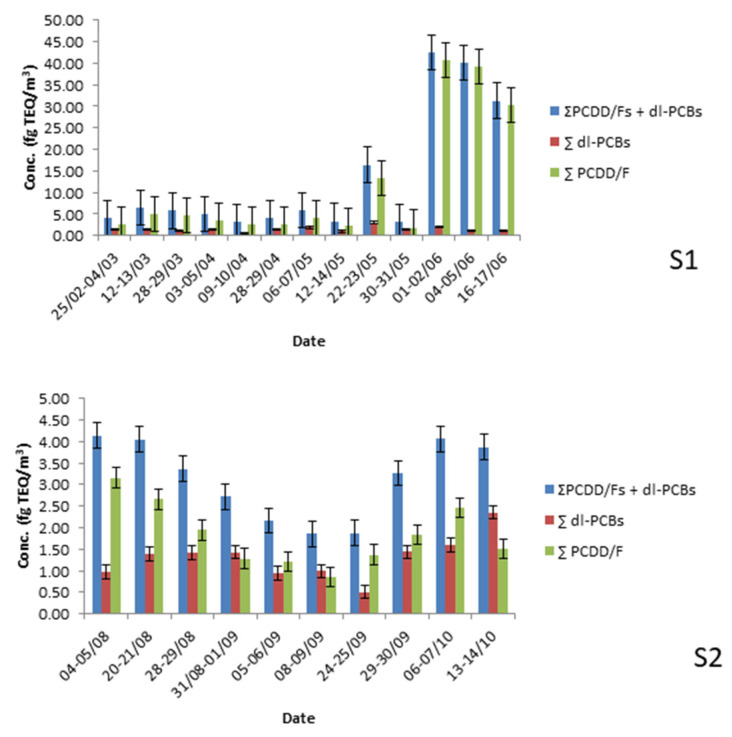
Dioxin and dl-PCB levels detected (fg TEQ/m^3^) at Chiva (**S1**) and Buñol (**S2**) stations.

Figure 2 and Table S3 show the results obtained in the S1 (Chiva) and S2 (Buñol) sampling sites for the total concentration (particulate phase plus gas phase) of the ∑dl-PCBs, ∑PCDD/Fs, and the sum of both, in fg TEQ/m^3^. Overall results are shown in Table 1.

Concentrations of dl-PCBs in Chiva (S1) ranged from 0.59 to 3.03 fg TEQ/m^3^, obtaining an average mean of 1.43 fg TEQ/m^3^. Obtained concentrations in Chiva for dl-PCBs were similar to other metallurgical and ceramic areas assessed in the Valencian Region in 2019 (López et al., 2021) [17] and lower than the obtained levels in two different urban areas (more than 2 fg TEQ/m^3^) of the city of Valencia (the big city close to the assessed sites, around 30 km). Moreover, levels of dl-PCBs in Chiva were lower than other industrial areas in Asia [31,32,33].

Concentrations of dl-PCBs in Buñol (S2) oscillated from 0.51 fg TEQ/m^3^ to 2.36 fg TEQ/m^3^, with an average mean of 1.31 fg TEQ/m^3^. The obtained average mean of dl-PCBs in this area was lower than the obtained levels in a previous study carried out by our research group in the surroundings of four industrial and two urban areas in the Valencian Region [17]. Furthermore, the obtained levels were around three to five times lower than other industrial areas in China [31], Vietnam [32], and Kuwait [33].

At the Chiva station (S1), levels of ∑PCDD/Fs ranged between 1.81 fg TEQ/m^3^ and 40.66 fg TEQ/m^3^, with an arithmetic mean of 11.75 fg TEQ/m^3^. Concentrations found at this station were higher than PCDD/Fs detected close to other cement industries in the Catalonia region of Spain [12,13,14]. In contrast, Rovira et al. (2011) [34] described higher levels of PCDD/Fs around the cement plant of Sant Feliu de Llobregat (34.2 fg TEQ/m^3^) in Spain. Furthermore, the range of PCDD/F concentrations detected at Chiva were similar than those founded in Turkey by Ercan and Dinçer (2016) [35] (between 0.03 and 27.9 fg TEQ/m^3^).

Concentrations of ∑PCDD/Fs ranged from 0.86 to 3.16 fg TEQ/m^3^, with an average mean of 1.68 fg TEQ/m^3^ at the Buñol site. PCDD/F levels were lower (2 to 20 times lower) than those detected close to other cement industries of Catalonia and Turkey (Table 2).

### 3.2. Results by Congeners

Table 3 shows the detection frequency and total concentrations (particulate phase plus gaseous phase) (fg/m^3^) calculated for each congener in the assessed samples.

In the case of the Chiva station (S1), the frequency of detection ranged from 8% (1,2,3,7,8-PCDD) to 100% (PCB-118, PCB-105, OCDF, and OCDD). The minimum concentration detected was 0.13 fg/m^3^ (1,2,3,4,7,8,9-HpCDF and 1,2,3,4,6,7,8-HpCDD), and the maximum was 2507.90 fg/m^3^ (PCB-118).

Buñol samples obtained frequencies of detection from 10% (PCB-114 and PCB-169) to 100% (PCB-118, 1,2,3,6,7,8-HxCDF, 2,3,4,6,7,8-HxCDF, 1,2,3,4,6,7,8-HpCDF, 1,2,3,4,6,7,8-HpCDD, and OCDD). The minimum concentration detected was 0.03 fg/m^3^ (1,2,3,4,7,8,9-HpCDF), and the maximum was 2899.53 fg/m^3^ (PCB-118).

The fg TEQ/m^3^ profile obtained for the dl-PCBs in Chiva and Buñol (see Figure S1) shows that PCB-126 is clearly the most present congener. These results are similar to those obtained in other industrial and urban areas of the Valencian Region [17] and other studies around the world [32,36,37]. According to Luthardt et al. (2002) [38], in industrial sources, the contributions of PCB-126 are more than 90% of PCB-TEQ. Moreover, the presence of PCB-169 is characteristic of industrial areas [39]. On the other hand, PCB-118 and PCB-156 are more characteristic of samples close to a cement kiln. Martínez et al. (2010) [39] showed that the concentration profiles taken at a cement plant stack present PCB-118 as the main PCB and PCB-105 as the second one.

For the profile concentrations (fg/m^3^) of PCDD/Fs in Chiva (see Figure S2) (fg/m^3^), the congeners with the highest concentrations were 1,2,3,4,7,8,9-HpCDF, OCDF, and OCDD. This profile is similar to those obtained by Abad et al. (2004) [40]. In contrast, at the Buñol station, the congeners with the highest concentrations were OCDD, 1,2,3,4,6,7,8-HpCDF and 1,2,3,4,6,7,8-HpCDD. López et al. (2021) [17] present a similar profile in the same region surrounding metallurgical factories, with OCDD, 1,2,3,4,6,7,8-HpCDD, and 1,2,3,4,6,7,8-HpCDF being the most prominent congeners.

### 3.3. Inhalation Risk Assessment

#### 3.3.1. Daily Inhalation Exposure

Table 4 shows the AM and percentile 95 (p95) values related to the inhalation exposure to dl-PCBs, PCDD/Fs, and the sum of dioxins, furans, and dl-PCBs in adults and children. To our knowledge, this is the first study to assess dl-PCB inhalation in the surroundings of cement plants.

**Figure 3:**
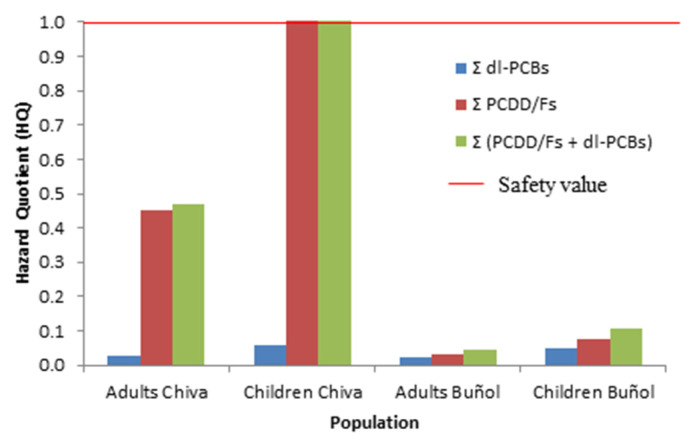
Hazard quotient at Chiva and Buñol stations for adults and children.

The inhalation exposure to dl-PCBs for adults at the Chiva station was 4.08 · 10^−4^ pg WHO TEQ kg^−1^ b.w. d^−1^, and 9.18 · 10^−4^ pg WHO TEQ kg^−1^ b.w. d^−1^ for children. Hazard quotients for dl-PCBs calculated for both populations were 2 times lower than 1 (see Figure 3), so no risk by inhalation pathway was observed. At the Buñol station, inhalation exposure to dl-PCBs in adults was 3.72 · 10^−4^ pg WHO TEQ kg^−1^ b.w. d^−1^, and 2.01 · 10^−3^ pg WHO TEQ kg^−1^ b.w. d^−1^ for children. The calculated HQ was lower than 1 (see Figure 3) in both populations, so no inhalation risk was noted. The studies carried out in the literature on the risk of dioxins and furans in the vicinity of cement plants did not estimate the risk of dl-PCBs, so it is not possible to compare the results obtained for dl-PCBs with other similar studies.

The inhalation exposure to adults of PCDD/Fs in Chiva was 3.34 · 10^−3^ pg WHO TEQ kg^−1^ b.w. d^−1^, and 7.52 · 10^−3^ pg WHO TEQ kg^−1^ b.w. d^−1^ for children. The calculated hazard quotient for children was slightly higher than 1 (1.02) at the Chiva station, considering p95 of daily inhalation exposure, so possible risk was observed due to inhalation. The obtained average value for inhalation exposure in adults is similar to other studies in the vicinity of cement plants, which range between 2.1 · 10^−3^ and 1.2 · 10^−2^ pg WHO TEQ kg^−1^ b.w. d^−1^ (see Table S4). The inhalation exposure to PCDD/Fs observed in Buñol (5.22 · 10^−4^ pg WHO TEQ kg^−1^ b.w. d^−1^ for adults and 1.17 · 10^−3^ pg WHO TEQ kg^−1^ b.w. d^−1^ for children) was a magnitude order lower than the studies carried out in Catalonia in the vicinity of cement plants (see Table 2). The calculated HQ for PCDD/Fs was lower than 1 in adults and children at the Buñol station.

#### 3.3.2. Chronic Exposure

**Figure 4:**
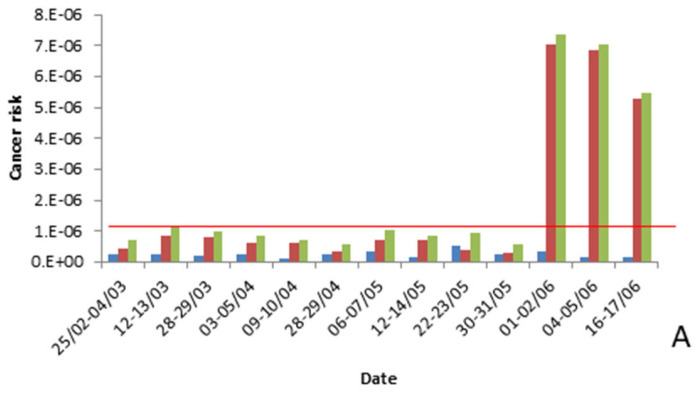

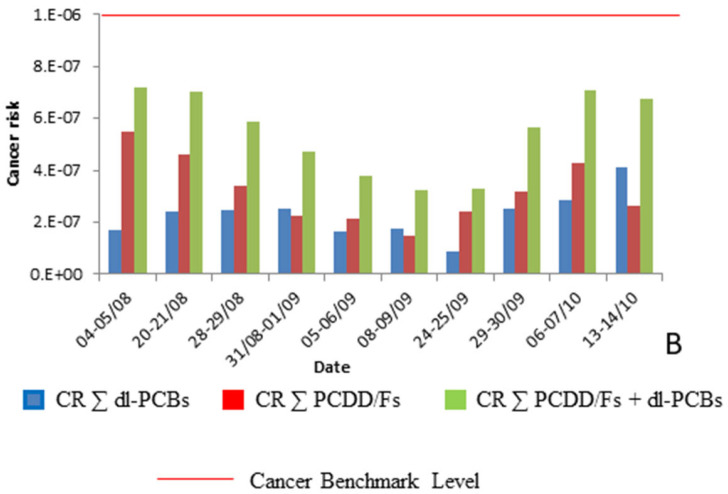
Cancer risk at Chiva (**A**) and Buñol stations (**B**).

The study assessed the chronic exposure to dioxins, furans, and dl-PCBs by calculating the cancer risk from inhalation at each station over a period of 70 years. The results indicate that certain samples from the Chiva sampling site (Figure 4A) show values higher than 10^−6^, especially those collected at the beginning of the summer. However, according to the EPA [27], cancer risk levels must be above 10^−4^ to be considered unacceptable, and chronic exposure levels in the area are lower than those detected in other industrial areas of the Valencian Region. On the other hand, chronic exposure levels at the Buñol station (Figure 4B) were below 10^−6^ in all samples evaluated, suggesting that the detected concentrations do not pose a long-term risk to the assessed population.

## 4. Study Limitations

Only the particle phase (PM_10_) has been collected and analyzed in the present study. Total concentrations including the gaseous phase have been calculated using a mathematical model previously validated by López et al., 2021 [17].

## 5. Conclusions

The concentrations of airborne dl-PCBs measured in the present study were found to be similar to those obtained in other industrial and urban areas in the assessed region. Regarding the obtained profiles for the analyzed compounds, dl-PCB profiles are identical at all studied sampling stations.

The estimated HQ for daily exposure in adults and children at the assessed sampling sites was less than 1 for dl-PCBs but slightly higher than 1 for PCDD/Fs in one of the investigated areas, indicating possible risk and the need of more-in-depth studies. For chronic exposure, some results at one sampling site have concerning levels. However, none of the obtained results showed exposure levels higher than 1.0 · 10^−4^; consequently, the adoption of immediate corrective measures is not required.

To our knowledge, this study is the first to assess inhalation risk in the population for dl-PCBs together with PCDDs and PCDFs in the vicinity of cement plants.

Taking into account our results, implementation of future monitoring programs in order to control industrial airborne pollution could be interesting for protecting the public health of the population living in the neighborhood of hotspot locations such as cement plants.

## Figures and Tables

**Table 1:** Overall concentrations (fg TEQ/m^3^) (particulate and gaseous phase) in ambient air samples at Chiva (n = 13) and Buñol (n = 10).

Analyte	AM ^1^	p50 ^2^	p95 ^3^	Min ^4^	Max ^5^
Chiva					
∑PCDD/Fs	11.75	4.07	39.75	1.81	40.66
∑dl-PCBs	1.43	1.42	2.33	0.59	3.03
∑PCDD/Fs + dl-PCBs	13.18	5.34	41.09	3.26	42.53
Buñol					
∑PCDD/Fs	1.83	1.68	2.94	0.86	3.16
∑dl-PCBs	1.31	1.41	2.02	0.51	2.36
∑PCDD/Fs + dl-PCBs	3.14	3.32	4.10	1.85	4.14

^1^ AM = arithmetic mean; ^2^ p50 = median; ^3^ p95 = percentile 95; ^4^ Min = minimum; ^5^ Max = maximum.

**Table 2:** PCDD/F air levels in the vicinity of cement plants in fg TEQ/m^3^.

Studies	Year	Place	AM ^1^	Min ^2^–Max ^3^
*Present study*	2020	Valencian Region (Spain)	1.83	0.86–3.16
	2020	Valencian Region (Spain)	11.75	1.81–40.66
[15]	2011	Catalonia (Spain)	7.0	4.7–8.6
	2012		7.0	6.8–8.3
[13]	2013	Catalonia (Spain)	9.0	8.0–11
	2015		8.0	5.0–10
[14]	2014	Catalonia (Spain)	30.0	10.0–50.0
[12]	2017	Catalonia (Spain)	4.0	3.0–5.0
[34]	2008	Catalonia (Spain)	17.1	5.9–40.7
	March 2008		13.8	4.9–29.0
	September 2009		34.2	16.1–46.8
[35]	2011	Istanbul (Turkey)	5.43	0.03–27.99
	2012		2.45	0.03–7.7

^1^ AM = arithmetic mean; ^2^ Min = minimum; ^3^ Max = maximum.

**Table 3:** Obtained results per congeners (fg/m^3^).

Analyte	*Chiva*			*Buñol*		
	DF ^1^ * (%)	AM ^2^ **	Range **	DF ^1^ * (%)	AM ^2^ **	Range **
PCB-81	39	56.71	33.51–100.52	60	60.32	33.51–100.52
PCB-77	92	61.17	4.06–100.08	90	49.63	2.70–78.44
PCB-123	69	115.63	5.74–269.65	80	99.83	11.47–212.28
PCB-118	100	1955.22	1415.87–2507.90	100	1509.26	677.81–2899.53
PCB-114	31	25.85	12.83–66.70	10	16.67	6.42–44.90
PCB-105	100	1232.27	907.59–1805.21	100	825.81	408.92–1476.08
PCB-126	23	12.27	4.09–28.64	20	11.05	4.09–20.46
PCB-167	85	48.23	2.26–86.02	80	89.87	4.53–373.50
PCB-157	31	17.72	3.49–59.33	60	49.21	6.98–177.98
PCB-156	92	95.87	3.33–156.63	90	135.30	6.66–403.23
PCB-169	23	2.66	1.38–5.52	10	3.31	1.38–9.67
PCB-189	54	8.51	3.03–22.74	70	20.77	4.55–53.05
2,3,7,8-TCDF	39	2.41	1.21–6.03	50	1.29	0.24–2.65
2,3,7,8-TCDD	-	0.93	0.93–0.93	-	0.29	0.29–0.29
1,2,3,7,8-PCDF	69	29.30	0.51–358.51	80	1.76	0.15–3.57
2,3,4,7,8-PCDF	39	1.76	0.42–5.83	70	1.19	0.12–2.50
1,2,3,7,8-PCDD	8	0.58	0.36–0.71	40	0.47	0.21–1.21
1,2,3,4,7,8-HxCDF	62	14.92	0.23–171.54	80	1.05	0.07–2.71
1,2,3,6,7,8-HxCDF	69	28.40	0.22–191.81	100	1.11	0.44–2.22
2,3,4,6,7,8-HxCDF	92	29.06	0.20–130.63	100	1.09	0.60–2.59
1,2,3,7,8,9-HxCDF	62	5.73	0.19–59.97	80	0.59	0.07–2.61
1,2,3,4,7,8-HxCDD	46	0.42	0.18–1.82	20	0.19	0.09–0.55
1,2,3,6,7,8-HxCDD	46	1.07	0.18–7.32	80	0.41	0.09–0.88
1,2,3,7,8,9-HxCDD	38	0.81	0.17–5.86	60	0.29	0.08–0.69
1,2,3,4,6,7,8-HpCDF	92	26.66	0.15–108.09	100	2.87	1.35–7.51
1,2,3,4,7,8,9-HpCDF	62	5.85	0.13–59.71	80	0.35	0.03–1.02
1,2,3,4,6,7,8-HpCDD	92	15.36	0.13–60.79	100	2.61	1.64–5.65
OCDF	100	15.28	0.87–64.13	90	1.30	0.04–4.37
OCDD	100	30.28	3.09–89.96	100	7.03	3.62–12.00
∑PCDD/F		208.82	15.76–897.92		23.88	15.75–40.00
∑dl-PCBs		3632.11	2727.57–4999.63		2871.02	1357.47–5616.43
∑(PCDD/F + dl-PCBs)		3840.94	2804.19–5551.62		2894.89	1374.29–5638.16

^1^ DF = detection frequency; ^2^ AM = arithmetic mean; * = In the particulate phase; ** = Total concentration (particulate phase plus gaseous phase).

**Table 4:** Daily inhalation exposure in adults (>12 years) and children (1.5–6 years) at each station in pg WHO TEQ kg^−1^ b.w. d^−1^.

Analyte	*Chiva*		*Buñol*	
	AM ^1^	p95 ^2^	AM ^1^	p95 ^2^
*Adults (>12 years)*				
Σ(PCDD/Fs+dl-PCBs)	3.75 · 10^−3^	1.17 · 10^−2^	8.93 · 10^−4^	1.17 · 10^−3^
Σ PCDD/Fs	3.34 · 10^−3^	1.13 · 10^−2^	5.22 · 10^−4^	8.35 · 10^−4^
Σ dl-PCBs	4.08 · 10^−4^	6.64 · 10^−4^	3.72 · 10^−4^	5.74 · 10^−4^
*Children (1.5–6 years)*				
Σ(PCDD/Fs+dl-PCBs)	8.44 · 10^−3^	2.63 · 10^−2^	2.01 · 10^−3^	2.63 · 10^−3^
Σ PCDD/Fs	7.52 · 10^−3^	2.54 · 10^−2^	1.17 · 10^−3^	1.88 · 10^−3^
Σ dl-PCBs	9.18 · 10^−4^	1.49 · 10^−3^	8.36 · 10^−4^	1.29 · 10^−3^

^1^ AM = arithmetic mean; ^2^ p95 = percentile 95.

## Data Availability

Not applicable.

## Supplementary Materials

The following supporting information can be downloaded at: https://www.mdpi.com/article/10.3390/toxics11040389/s1, Section S1: Analytical performance parameters and identification criteria; Table S1: Sampling dates of the analyzed filters and total sample volume (m^3^); Table S2: Inhalation Unit risk (µg/m^3^)^−1^ and Slope Factor ${\left(\frac{\mathrm{mg}}{{\mathrm{kg}\;\mathrm{day}}^{-1}}\right)}^{-1};\;$Table S3: Concentrations of ΣPCDD/Fs + dl-PCBs, ∑ dl-PCBs, and ∑ PCDD/Fs obtained at Chiva (C) and Buñol (B) stations in fg TEQ/m^3^; Table S4: Exposure of dioxins and furans in adults in the vicinity of cement plants; Figure S1: Profile of concentrations of dl-PCBs detected at Chiva and Buñol stations using concentrations (fg/m^3^) and toxic equivalents (TEQ) (fg TEQ/m^3^); Figure S2: Profile of concentrations of PCDD/Fs detected at Chiva and Buñol stations using concentrations (fg/m^3^) and toxic equivalents (TEQ) (fg TEQ/m^3^).
