# Supplementary material for: Exposure and Risk Assessment to Airborne dl-PCBs and Dioxins in the Population Living in the Neighborhood of a Cement Plant: A Pilot Study in the Valencian Region of Spain
Source: Toxics. 2023 Apr 20;11(4):389. doi: 10.3390/toxics11040389 (PMC10143573; doi:10.3390/toxics11040389)
Supplement: Supplementary file 1 [file toxics-11-00389-s001.zip › toxics-2326786-supplementary.pdf]

Section S-1. Analytical performance parameters and identification criteria

To identify and confirm the analyzed compounds, the criteria described in Commission Regulation 2017/771 (EU, 2017) and EPA method 1613 (USEPA, 2014) have been used:

a) The absolute retention time obtained in the verification test for internal standards $^{13}C_{12}$-1,2,3,4-TCDD and $^{13}C_{12}$-1,2,3,7,8,9-HxCDD must not exceed ± 15 seconds with respect to retention times obtained in the calibration curve.

b) The ratio of the ions (qualifier and quantifier) must not show a difference of more than 15% with respect to the theoretical values.

c) The resolution of the instrument maintained at 10,000 (10% valley).

d) The standard deviation of the relative response factors of the response factors should be used to evaluate each calibration curve.

e) Spiked samples at three different concentration levels must be used as quality control of the native compounds (dioxins and dl-PCB). A 20% difference in recoveries will be considered acceptable.

f) The range of recoveries of the surrogates (EPA 1613-LCS and WP-LCS) should be between 60-120 %, except for OCDD (50-100 %).

Table S1. Sampling dates of the analyzed filters and total sample volume (m^3).

Estación	Date filter 1	Date filter 2	Total volume (m^3)	Code
Chiva	25/02/2020	04/03/2020	1473.31	C1
	12/03/2020	13/03/2020	1453.25	C2
	28/03/2020	29/03/2020	1448.20	C3
	03/04/2020	05/04/2020	1444.14	C4
	09/04/2020	10/04/2020	1453.94	C5
	28/04/2020	29/04/2020	1460.58	C6
	06/05/2020	07/05/2020	1466.30	C7
	12/05/2020	14/05/2020	1457.44	C8
	22/05/2020	23/05/2020	1471.85	C9
	30/05/2020	31/05/2020	1470.17	C10
	01/06/2020	02/06/2020	1471.85	C11
	04/06/2020	05/06/2020	1469.78	C12
	16/06/2020	17/06/2020	1469.14	C13
Buñol	04/08/2020	05/08/2020	1454.05	B1
	20/08/2020	21/08/2020	1460.07	B2
	28/08/2020	29/08/2020	1457.37	B3
	31/08/2020	01/09/2020	1443.88	B4
	05/09/2020	06/09/2020	1443.88	B5
	08/09/2020	09/09/2020	1435.79	B6
	24/09/2020	25/09/2020	1440.51	B7
	29/09/2020	30/09/2020	1436.46	B8
	06/10/2020	07/10/2020	1439.88	B9
	13/10/2020	14/10/2020	1438.70	B10

Table S2. Inhalation Unit risk $(\mu g/m^3)^{-1}$ and Slope Factor $\left(\frac{mg}{kg\ day^{-1}}\right)^{-1}$

Analyte	IUR $(\mu g/m^3)^{-1}$	SF $(mg/kg\ day^{-1})^{-1}$
PCB-81	0.0110	38.5
PCB-77	0.0038	13.3
PCB-123	0.0011	3.85
PCB-118	0.0011	3.85
PCB-114	0.0011	3.85
PCB-105	0.0011	3.85
PCB-126	38.000	13300
PCB-167	0.0011	3.85
PCB-157	0.0011	3.85
PCB-156	0.0011	3.85
PCB-169	11.000	3850
PCB-189	0.0011	3.85
2,3,7,8-TCDF	38.000	13300
2,3,7,8-TCDD	380.000	133000
1,2,3,7,8-PCDF	11.000	3850
2,3,4,7,8-PCDF	110.000	38500
1,2,3,7,8-PCDD	380.000	133000
1,2,3,4,7,8-HxCDF	38.000	13300
1,2,3,6,7,8-HxCDF	38.000	13300
2,3,4,6,7,8-HxCDF	38.000	13300
1,2,3,7,8,9-HxCDF	38.000	13300
1,2,3,4,7,8-HxCDD	38.000	13300
1,2,3,6,7,8-HxCDD	38.000	13300
1,2,3,7,8,9-HxCDD	38.000	13300
1,2,3,4,6,7,8-HpCDF	0.3800	1330
1,2,3,4,7,8,9-HpCDF	0.3800	1330
1,2,3,4,6,7,8-HpCDD	0.3800	1330
OCDF	0.0110	38.5
OCDD	0.0110	38.5

Table S3. Concentrations of ΣPCDD / Fs + dl-PCBs, ∑ dl-PCBs and ∑ PCDD / Fs obtained at Chiva (C) and Buñol (B) stations in fg TEQ / m^3

**Muestras**	**∑ dl-PCBs**	**∑ PCDD/Fs**	**Σ (PCDD/Fs + dl-PCBs)**
**C1 (25/02-04/03)**	1.45	2.62	4.06
**C2 (12-13/03)**	1.44	4.98	6.43
**C3 (28-29/03)**	1.15	4.58	5.74
**C4 (03-05/04)**	1.42	3.51	4.93
**C5 (09-10/04)**	0.59	2.68	3.26
**C6 (28-29/04)**	1.42	2.68	4.10
**C7 (06-07/05)**	1.85	4.07	5.92
**C8 (12-14/05)**	0.96	2.34	3.30
**C9 (22-23/05)**	3.03	13.38	16.41
**C10 (30-31/05)**	1.46	1.81	3.26
**C11 (01-02/06)**	1.87	40.66	42.53
**C12 (04-05/06)**	0.99	39.15	40.13
**C13 (16-17/06)**	1.02	30.26	31.28
**B1 (04-05/08)**	0.98	3.16	4.14
**B2 (20-21/08)**	1.39	2.66	4.05
**B3 (28-29/08)**	1.42	1.95	3.36
**B4 (31/08-01/09)**	1.43	1.29	2.72
**B5 (05-06/09)**	0.95	1.22	2.17
**B6 (08-09/09)**	0.99	0.86	1.85
**B7 (24-25/09)**	0.51	1.37	1.88
**B8 (29-30/09)**	1.44	1.83	3.27
**B9 (06-07/10)**	1.60	2.47	4.06
**B10 (13/14-10)**	2.36	1.52	3.87

Table S4. Exposure of dioxins and furans in adults in the vicinity of cement plants			
**Studies**	**Year**	**Country**	**DID (pg WHO TEQ kg^{-1} b.w. day^{-1})**
Rovira et al. 2015	2011	Sta. Margarida i els Monjos, Catalonia	$2.5*10^{-3}$
	2012		$2.6*10^{-3}$
Mari et al. 2017	2013	Sant Vicenç dels Horts, Catalonia	NA
	2015		$2.1*10^{-3}$
Rovira et al. 2016	2014	Montcada, Catalonia	$1.2*10^{-2}$
Mari et al. 2018	2017	Alcanar, Catalonia	$2.8*10^{-3}$

NA= Not available

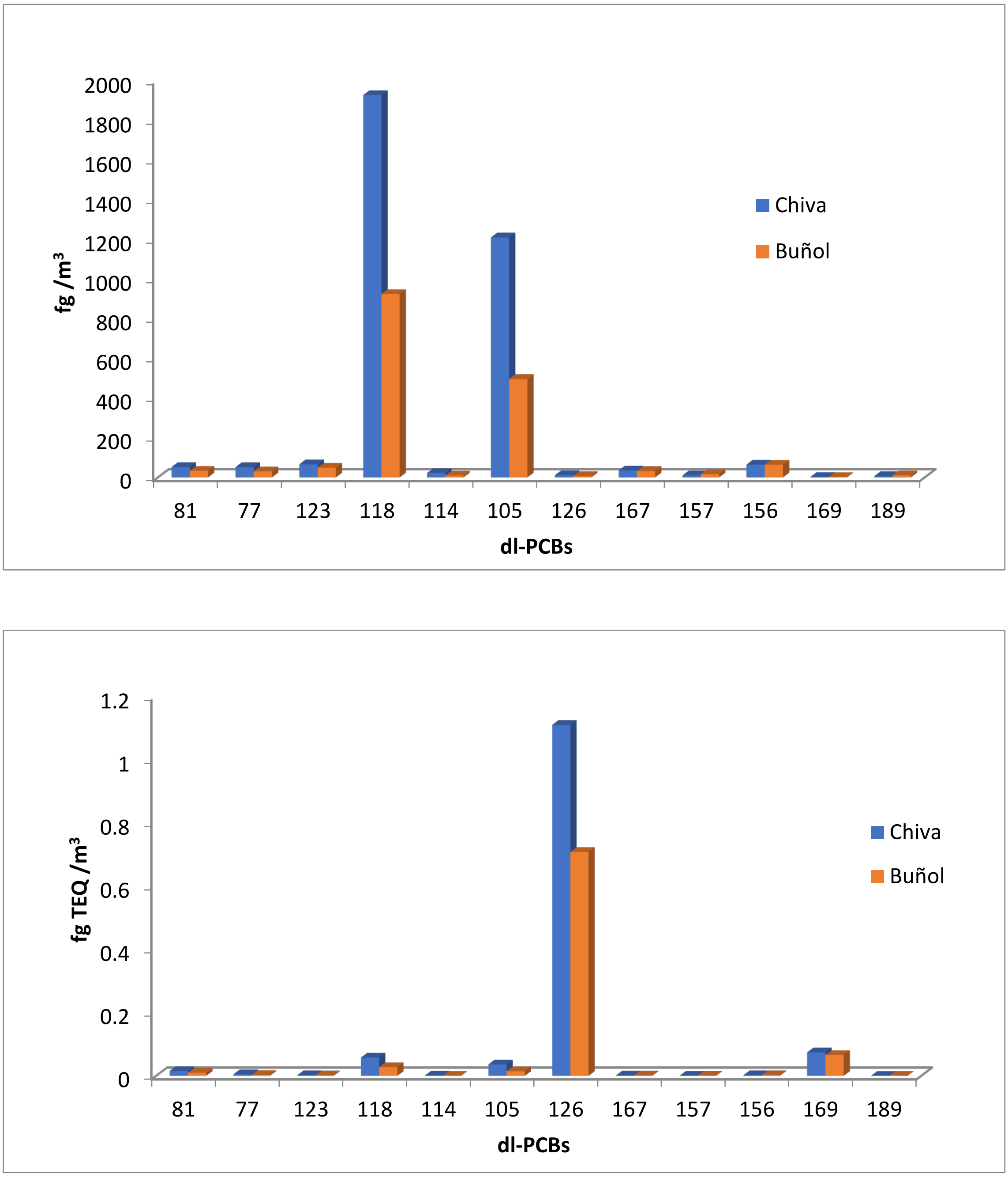

Figure S1. Profile of concentrations of dl-PCBs detected at Chiva and Buñol stations using concentrations (fg / m^3) and toxic equivalents (TEQ) (fg TEQ /m^3).

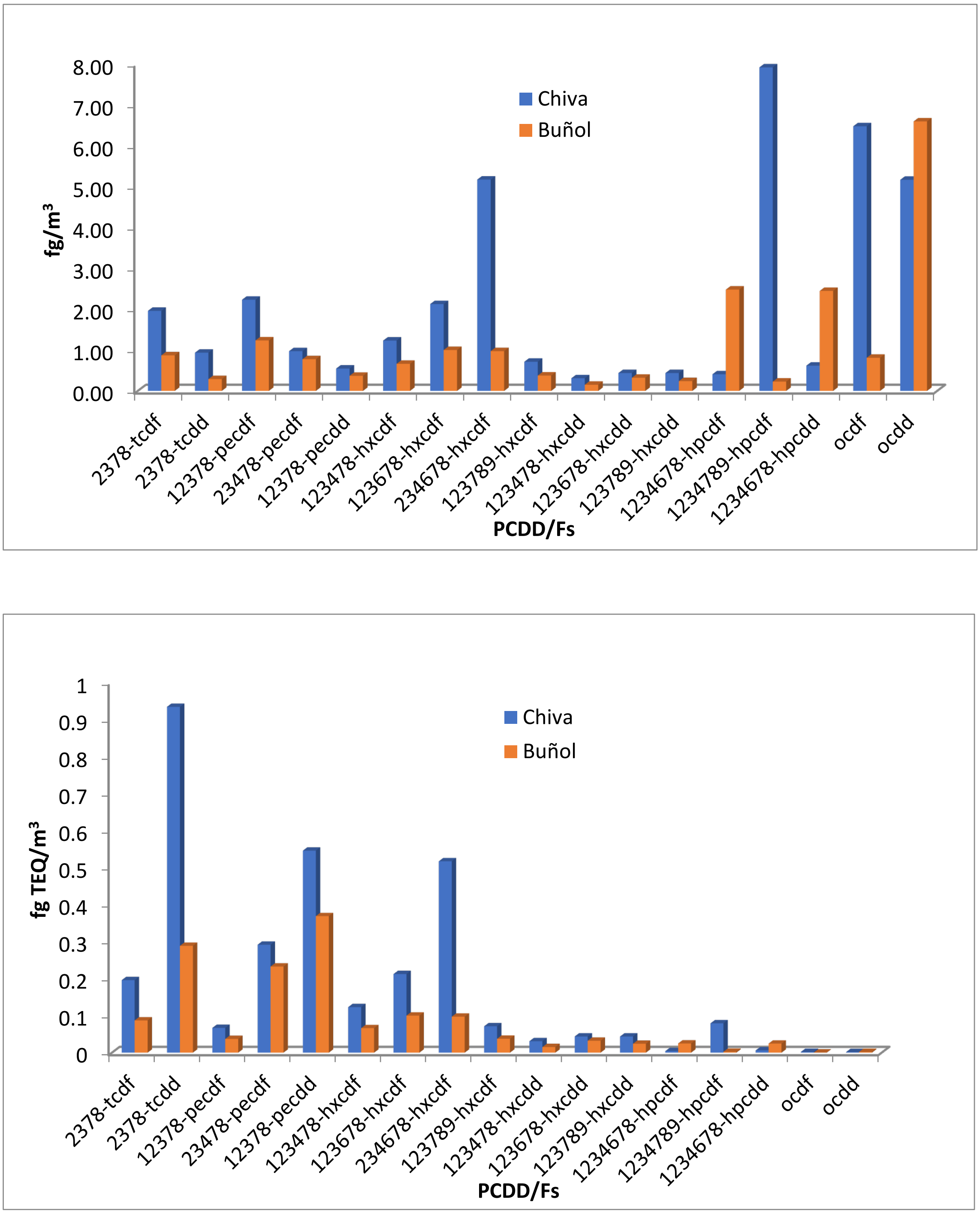

Figure S2. Profile of concentrations of PCDD/Fs detected at Chiva and Buñol stations using concentrations (fg / m^3) and toxic equivalents (TEQ) (fg TEQ / m^3).
